# Unmasking Subclinical Atherosclerosis: The Impact of Carotid Ultrasound on the Evaluation of Patients With Rheumatoid Arthritis

**DOI:** 10.7759/cureus.80525

**Published:** 2025-03-13

**Authors:** Ramiro A Moreno, Cesar Ayala Ugarte, Emmanuel Amaro Hernández, Eric Sánchez Olivan, Daniel I Leal Villanueva, Fabiola Flores Serrano, Diana Laura Rojas de León, Azul F Zaragoza Pérez, Angie Pamela Reyes Loaiza, Fernanda J Velázquez Domínguez, Cesar E Ibarra Hernández, María de la Luz Flores Reséndiz, Jaqueline L Castillo, Jose R Flores Valdés

**Affiliations:** 1 Cardiology, Autonomous University of Nuevo León, Monterrey, MEX; 2 Cardiology, Autonomous University of Guadalajara, Guadalajara, MEX; 3 General Medicine, Monterrey Institute of Technology and Higher Education, Monterrey, MEX; 4 Cardiology, National Autonomous University of Mexico, Mexico, MEX; 5 General Medicine, Michoacan University of Saint Nicholas of Hidalgo, Morelia, MEX; 6 General Medicine, Autonomous University of Guadalajara, Guadalajara, MEX; 7 General Medicine, Monterrey Institute of Technology and Higher Education, Mexico, MEX; 8 General Medicine, Siglo XXI University Campus, Apodaca, MEX; 9 General Medicine, Technical University of Machala, Machala, ECU; 10 General Medicine, Autonomous University of the State of Morelos, Cuernavaca, MEX; 11 General Medicine, University of Guadalajara, Guadalajara, MEX; 12 General Medicine, University of the Valley of Mexico, Saltillo, MEX; 13 General Practice, Oncology Consultants, Houston, USA

**Keywords:** arterial stiffness, atherosclerosis, cardiovascular risk, carotid ultrasound, endothelial dysfunction, inflammation, intima-media thickness, rheumatoid arthritis, subclinical atherosclerosis, ultrasonography

## Abstract

Rheumatoid arthritis (RA) is the most prevalent autoimmune disease, characterized by chronic joint inflammation and systemic symptoms, particularly affecting cardiovascular health through atherosclerosis development. This study investigates the role of carotid ultrasound in detecting atherosclerosis in RA patients by measuring carotid intima-media thickness (CIMT). We conducted a comprehensive review of studies published from 2017 to 2024, sourced from PubMed, focusing specifically on CIMT measurements in RA patients. Our analysis included eight studies involving a total of 1,242 participants. The results demonstrate that increased CIMT measurements in RA patients are associated with a higher risk of subclinical atherosclerosis. Notably, most studies (N = 7) reported statistically significant increases in CIMT, while only one study found no significant difference. These findings suggest that CIMT is a valuable tool for assessing cardiovascular risk in RA patients, advocating for the more frequent use of carotid ultrasound as a noninvasive clinical assessment method for this population. However, further multicentric randomized controlled trials and enhanced training for healthcare providers are necessary to ensure accurate CIMT measurements in routine practice.

## Introduction and background

Rheumatoid arthritis (RA) is the most common form of autoimmune arthritis and a significant public health challenge, with studies suggesting that its prevalence may be increasing [1]. This chronic, systemic inflammatory condition primarily affects the joints and periarticular soft tissues, causing both painful inflammatory arthritis and substantial complications beyond the joints themselves [2,3]. Moreover, the impact of RA goes far beyond these immediate symptoms. It has been shown to have systemic implications, particularly in the cardiovascular system. In fact, RA is even considered an independent risk factor for developing cardiovascular pathology [4,5].

Chronic inflammation, which is a hallmark of RA [6], not only contributes to joint damage but also accelerates the development of atherosclerosis, a critical factor in the onset of coronary artery disease [7]. Patients with RA exhibit a higher prevalence of atherosclerotic carotid plaques, which correlates with disease duration and reflects the widespread nature of atherosclerosis in this population [8].

Studies further highlight that individuals with RA face a markedly increased mortality risk, with rates considerably higher than those observed in the general population. In particular, cardiovascular disease (CVD) is a significant contributor to this increased mortality, accounting for CVD as the primary cause of death among RA patients [9]. This excess risk translates into a reduction in life expectancy of an average of six to seven years [10]. To put it in perspective, the standardized mortality ratio for cardiovascular death in RA patients is estimated to be between 1 and 2 (95% CI: 1.05-1.43), pointing to a substantial burden borne by this population according to van den Hoek et al. [9].

**Figure 1:**
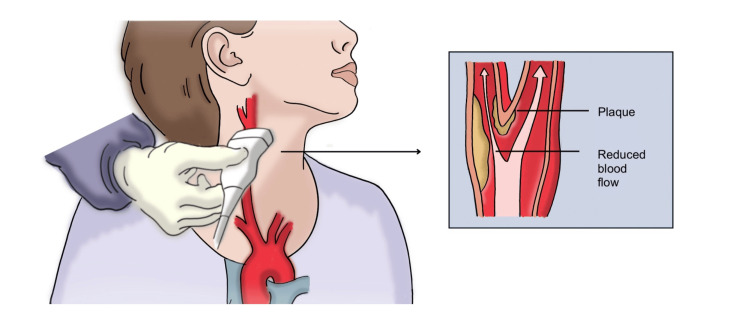
Schematic representation of carotid ultrasound The left side illustrates the ultrasound procedure, where a transducer is applied to the carotid artery. The right side provides a cross-sectional view of the artery, highlighting atherosclerotic plaque formation and the resulting reduction in blood flow. Carotid ultrasound is a noninvasive imaging technique used to detect subclinical atherosclerosis and assess vascular health in patients at increased cardiovascular risk. Image credit: Ramiro A. Moreno

In recent years, carotid ultrasound has been employed as a valuable noninvasive tool measuring carotid intima-media thickness (CIMT; Figure 1). These measurements provide a quantitative method for assessing CVD risk and tracking changes in disease status over time, making them particularly useful in clinical and research settings [5,11]. While a meta-analysis has shown that adding CIMT measurements to traditional risk scores, such as the Framingham Risk Score, only slightly improves 10-year risk prediction for myocardial infarctions or strokes [12]. Another meta-analysis indicates that interventions reducing CIMT progression are associated with a significant reduction in CVD risk [13].

As a result, there is still ongoing debate about the routine use of CIMT in assessing cardiovascular risk, particularly among RA patients. Nonetheless, the European League Against Rheumatism (EULAR) has presented guidelines recommending carotid ultrasound for screening of asymptomatic atherosclerotic plaques as part of a comprehensive CVD risk assessment, especially given the high pretest probability of plaque detection in this population [14].

This systematic review aims to explore the utility of carotid ultrasound in the detection of subclinical atherosclerosis in patients with RA and to assess its potential role in refining cardiovascular risk stratification and clinical management. By examining these aspects, the review aims to clarify whether carotid ultrasound could lead to earlier interventions, improving outcomes and quality of life for people living with RA.

## Review

Methods

The present systematic review adheres to the guidelines presented by Preferred Reporting Items for Systematic reviews and Meta-Analyses (PRISMA) 2020 [15,16].

Searching Methods

To ensure the selection of high-quality studies, strict inclusion and exclusion criteria were implemented. The exclusion process was meticulously carried out to maintain the relevance and integrity of the studies reviewed. Research that did not specifically investigate the role of carotid ultrasound in identifying subclinical atherosclerosis in patients with RA was omitted. Furthermore, studies that were not accessible in full-text format were not included in the analysis.

The literature review was conducted solely using the PubMed database (Table 1). A PRISMA flowchart was used to guide the article selection process, ensuring the formation of a consistent dataset and improving the precision and reliability of the findings.

**Table 1:** PubMed search strategy and results

Search strategy	Results
("ultrasonography, carotid arteries"[MeSH Terms] OR ("ultrasonography"[All Fields] AND "carotid"[All Fields] AND "arteries"[All Fields]) OR "carotid arteries ultrasonography"[All Fields] OR ("carotid"[All Fields] AND "ultrasound"[All Fields]) OR "carotid ultrasound"[All Fields] OR ("ultrasonography, carotid arteries"[MeSH Terms] OR ("ultrasonography"[All Fields] AND "carotid"[All Fields] AND "arteries"[All Fields]) OR "carotid arteries ultrasonography"[All Fields] OR ("carotid"[All Fields] AND "artery"[All Fields] AND "ultrasound"[All Fields]) OR "carotid artery ultrasound"[All Fields]) OR ("carotid intima media thickness"[MeSH Terms] OR ("carotid"[All Fields] AND "intima media"[All Fields] AND "thickness"[All Fields]) OR "carotid intima media thickness"[All Fields] OR ("carotid"[All Fields] AND "intima"[All Fields] AND "media"[All Fields] AND "thickness"[All Fields]) OR "carotid intima media thickness"[All Fields]) OR ("ultrasonography, doppler"[MeSH Terms] OR ("ultrasonography"[All Fields] AND "doppler"[All Fields]) OR "doppler ultrasonography"[All Fields] OR ("doppler"[All Fields] AND "ultrasound"[All Fields]) OR "doppler ultrasound"[All Fields]) OR ("diagnostic imaging"[MeSH Subheading] OR ("diagnostic"[All Fields] AND "imaging"[All Fields]) OR "diagnostic imaging"[All Fields] OR "ultrasonography"[All Fields] OR "ultrasonography"[MeSH Terms] OR "ultrasonographies"[All Fields])) AND (("arthritis, rheumatoid"[MeSH Terms] OR ("arthritis"[All Fields] AND "rheumatoid"[All Fields]) OR "rheumatoid arthritis"[All Fields] OR ("rheumatoid"[All Fields] AND "arthritis"[All Fields])) AND ((("subclinic"[All Fields] OR "subclinical"[All Fields] OR "subclinically"[All Fields] OR "subclinicals"[All Fields]) AND ("atherosclerosis"[MeSH Terms] OR "atherosclerosis"[All Fields] OR "atheroscleroses"[All Fields])) OR ("Early"[All Fields] AND ("atherosclerosis"[MeSH Terms] OR "atherosclerosis"[All Fields] OR "atheroscleroses"[All Fields])) OR ("plaque, atherosclerotic"[MeSH Terms] OR ("plaque"[All Fields] AND "atherosclerotic"[All Fields]) OR "atherosclerotic plaque"[All Fields] OR ("atherosclerotic"[All Fields] AND "plaque"[All Fields])) OR ("carotid artery diseases"[MeSH Terms] OR ("carotid"[All Fields] AND "artery"[All Fields] AND "diseases"[All Fields]) OR "carotid artery diseases"[All Fields] OR ("carotid"[All Fields] AND "atherosclerosis"[All Fields]) OR "carotid atherosclerosis"[All Fields]) OR (("paroi arterielle"[Journal] OR ("arterial"[All Fields] AND "wall"[All Fields]) OR "arterial wall"[All Fields]) AND ("thicken"[All Fields] OR "thickened"[All Fields] OR "thickening"[All Fields] OR "thickenings"[All Fields] OR "thickens"[All Fields]))))	985

Types of Studies

To explore the role of carotid ultrasound in identifying subclinical atherosclerosis in patients with RA, we conducted a systematic review of studies published between 2017 and 2024. Our analysis focused on observational research, including cross-sectional and case-control studies. We selected studies that involved patients with a confirmed diagnosis of RA, where carotid ultrasound was used to assess CIMT.

Certain types of publications were excluded from this review, including case reports, letters to the editor, case series, book chapters, conference abstracts, editorials, and news articles. Studies were also excluded if they involved patients with RA who had a prior history of CVD, lacked a confirmed RA diagnosis, or did not employ carotid ultrasound for assessment. Furthermore, any study where essential data could not be retrieved was omitted.

Types of Participants

This review applied strict selection criteria, focusing on individuals between 40 and 75 years of age. We included female patients who met either the 2010 American College of Rheumatology (ACR)/EULAR classification criteria or the 1987 ACR criteria for RA diagnosis.

Exclusion criteria encompassed patients with comorbidities such as diabetes mellitus, hypertension, chronic kidney disease (defined as an estimated glomerular filtration rate below 59 mL/min/1.73 m²), coronary artery disease, cerebrovascular disease, hyperuricemia, and peripheral vascular disease. Additionally, individuals with RA who had preexisting atherosclerotic CVD, those diagnosed with mixed connective tissue disease, and pregnant women were not included in the review.

Type of Intervention

For inclusion, studies needed to compare CIMT measurements between RA patients and a control group of individuals without the disease.

Type of Outcomes

This review considered studies that met the predefined criteria to evaluate the effectiveness of carotid ultrasound in assessing cardiovascular risk in RA patients. The primary objective was to analyze CIMT as a quantitative marker for early detection of cardiovascular conditions such as asymptomatic atherosclerosis and carotid artery wall hypertrophy. Additionally, we aimed to determine whether integrating this imaging technique into routine clinical practice could enhance existing cardiovascular risk management strategies in RA patients.

Selection of Studies, Data Extraction, and Screening

To ensure a rigorous selection process, two reviewers (CAU and AFZP) independently screened article titles and abstracts using Rayyan [17], a web-based tool designed for systematic reviews. A third independent reviewer (RAMM) then verified the relevance of the studies based on predefined inclusion and exclusion criteria. After this initial screening, a comprehensive full-text evaluation was conducted. In this phase, two additional reviewers (RAMM and FFS) independently assessed the studies, applying the same selection criteria. Any disagreements during this process were resolved through discussion, with the involvement of a third reviewer (CAU) when necessary to reach a final consensus.

All studies retrieved from the search were carefully examined for relevance. Those considered potentially eligible underwent a full evaluation against the inclusion and exclusion criteria before being either selected for inclusion or excluded from further analysis.

For each included study, data extraction was performed systematically, capturing essential information such as the study title, publication year, study duration, research design, population characteristics (e.g., age, gender, and disease duration), methodology for carotid ultrasound assessment (e.g., intima-media thickness measurement and plaque detection), primary outcomes assessed, secondary outcomes (if applicable), and key findings regarding cardiovascular risk in RA patients.

Assessment of Risk of Bias in Included Studies

To evaluate the reliability and methodological rigor of the selected studies, we adhered to the guidelines established in the Cochrane Handbook for Systematic Reviews of Interventions [18]. The Newcastle-Ottawa Scale (NOS) was applied to assess the quality of case-control and cohort studies. Two independent reviewers conducted a detailed assessment of the risk of bias in each study, meticulously following the criteria and guidelines of the designated evaluation tools. Any differences in judgment or conflicting conclusions were addressed through discussion, with a third, blinded reviewer (DILV) assisting in reaching a final decision when necessary.

Based on the Cochrane Handbook and NOS guidelines, studies were classified into three categories regarding their risk of bias: low, high, or unclear. Additionally, any modifications in the quality of evidence, whether they involved upgrading or downgrading, were explicitly documented in the summary of findings table. Each bias assessment was accompanied by a transparent justification, ensuring clarity in the evaluation process.

By maintaining a structured and meticulous approach, this study aimed to ensure the inclusion of high-quality research while minimizing potential sources of bias.

Results

We conducted a systematic literature review to assess the effectiveness of carotid ultrasound in evaluating subclinical atherosclerosis in patients with RA. Our search spanned from 2017 to 2024, covering articles indexed in the PubMed database. We employed a combination of keywords related to “rheumatoid arthritis”, “subclinical atherosclerosis”, “carotid ultrasound”, “atherosclerosis”, and “intima-media thickness”.

**Figure 2:**
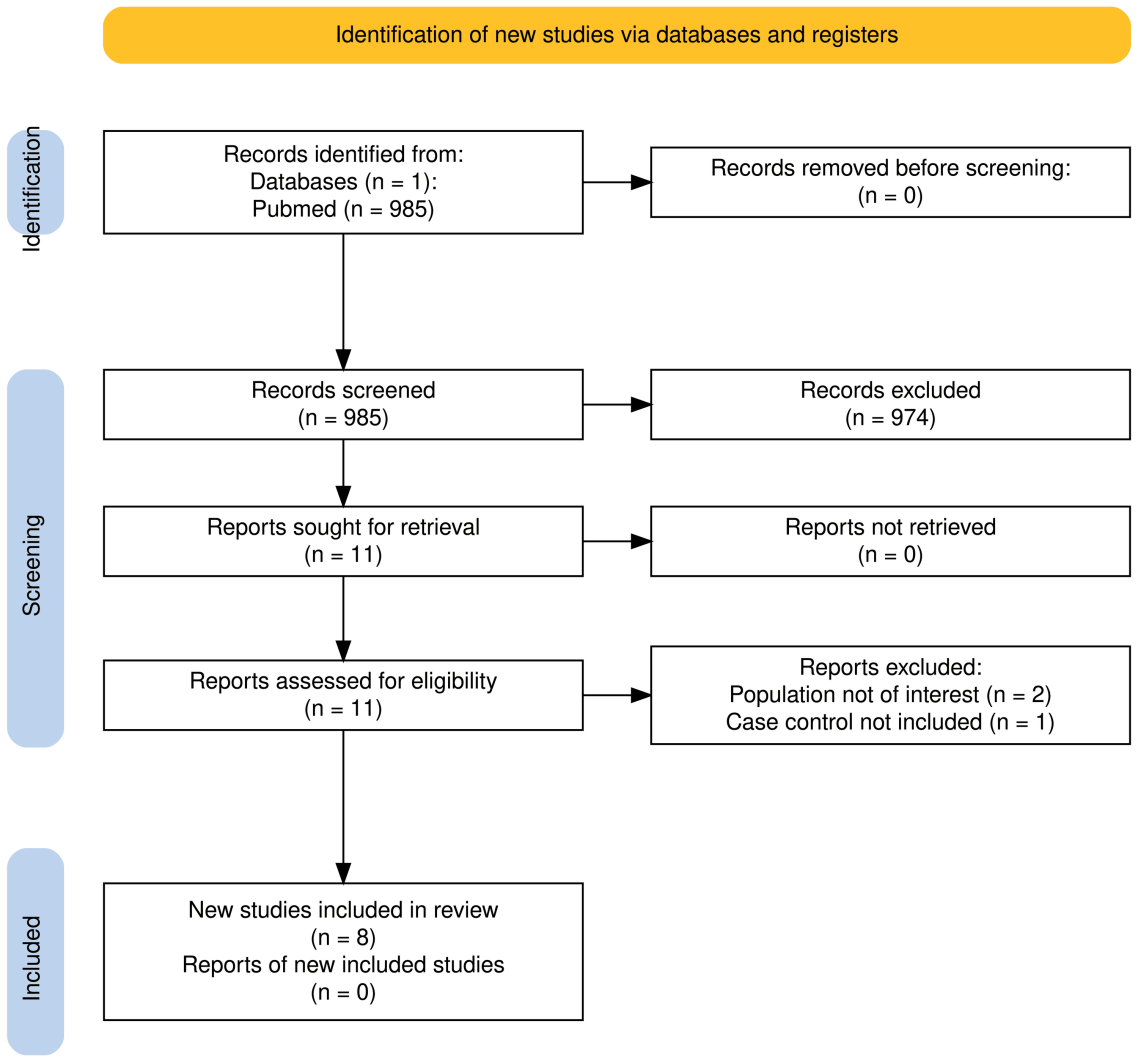
PRISMA flowchart The PRISMA flow diagram outlines the process of study selection for a systematic review. A total of 985 records were identified from PubMed (n = 985) and no records from registers (n = 0). After screening, 974 records were excluded based on predefined criteria. Of the 11 records sought for retrieval, all were retrieved (n = 0 reports not retrieved). Finally, 11 reports were assessed for eligibility, of which three were excluded due to reasons such as population not of interest (n = 2) and case-control not included (n = 1). Ultimately, eight studies were included in the review. PRISMA, Preferred Reporting Items for Systematic reviews and Meta-Analyses

Outlined in the PRISMA chart [19], a total of 985 studies were retrieved, of which 11 underwent full-text analysis. Ultimately, eight articles were deemed eligible after removing duplicates, screening titles and abstracts, and excluding nonaccessible information, as illustrated in Figure 2.

The systematic review includes a total of eight articles, of which 87.5% (n = 7) are cross-sectional/case-control studies and 12.5% (n = 1) is a cohort study. The studies were conducted in various countries, including 25% (n = 2) in Mexico, 12.5% (n = 1) in Slovenia, 12.5% (n = 1) in Iran, 12.5% (n = 1) in Poland, 12.5% (n = 1) in India, 12.5% (n = 1) in Pakistan, and 12.5% (n = 1) in Russia. The total number of patients evaluated across all included articles is 1,297, of which 758 were patients with RA and 539 were healthy controls. All the patients underwent carotid ultrasound. The range of participants per study varies from 31 to 275. The age range of the included participants spans from 42 to 56 years. Both male and female participants were evaluated. The follow-up period ranged from three years to 15 years.

For the risk of bias assessment, the studies were evaluated using the Quality Assessment Scale [18]. A total of eight studies were evaluated. All of the cohort and case-control studies were classified as “Good quality” (n = 8). The details of the assessment for each study are presented in Table 2.

**Table 2:** NOS ROB The NOS for RoB assesses the methodological quality of observational studies based on three domains: selection (0-4 points), comparability (0-2 points), and outcome/exposure (0-3 points). The total score ranges from 0 to 9, with higher scores indicating a lower risk of bias. Studies scoring 7-9 points are considered “Good” quality, 4-6 points “Fair,” and ≤3 points “Poor.” This evaluation provides a standardized approach to assess the reliability of included studies. NOS, Newcastle-Ottawa Scale; RoB, Risk of Bias

Author and year	Study design	Selection	Comparability	Outcome/exposure	Total	Subjective evaluation
Koren Krajnc et al. (2021) [20]	Prospective cohort	4	1	3	8	Good
Rajabzadeh et al. (2023) [21]	Cross-sectional case-control	3	1	3	7	Good
Targońska-Stȩpniak et al. (2019) [22]	Cross-sectional case-control	3	1	3	7	Good
Galarza-Delgado et al. (2023) [23]	Cross-sectional case-control	4	1	3	8	Good
Arora et al. (2022) [24]	Cross-sectional case-control	4	1	3	8	Good
Khaliq et al. (2023) [25]	Cross-sectional case-control	4	1	3	8	Good
Gerasimova et al. (2023) [26]	Cross-sectional case-control	4	1	3	8	Good
Wah-Suarez et al. (2019) [27]	Cross-sectional observational	4	1	3	8	Good

General Outcomes

The findings shown in Table 3 provide insight into the relation between RA and CIMT measure and plaque percentage, incorporating data from 2018 to 2023 across various countries, and were mostly conducted in rheumatology units. Patients had a mean age ranging from 42 to 57 years. As for CIMT values, across all studies, RA patients exhibited higher CIMT compared to control groups, even early-stage RA patients showed significant vascular changes. Among studies that highlighted sex differences in RA, male RA patients tend to have an increased CIMT measure in comparison to female and control patients; however, chronic systemic inflammation appears to be the most impactful driver of atherosclerosis formation in RA patients, rather than traditional cardiovascular risk factors, highlighted by the increase in CRP levels and altered lipid profiles among RA.

**Table 3:** General outcomes CG, control group; CIMT, carotid intima-media thickness; CVD, cardiovascular disease; LDL, low-density lipoprotein; RA, rheumatoid arthritis; RC, right carotid

Author and year	Study design	Country	Patients	Mean age (RA)	CIMT (mm)	Plaque % (RA)	Plaque % (CG)	Key points
Koren Krajnc et al. (2021) [20]	Prospective cohort	Slovenia	RA: 60; CG: 34	57 ± 5.5	RA: 0.688 ± 0.108; CG: 0.604	42.4% (N = 25)	12.9% (N = 4)	In young premenopausal females with RA, inflammation risk factors appear to be more significant than conventional risk factors for the atherosclerotic process.
Rajabzadeh et al. (2023) [21]	Cross-sectional case-control	Iran	RA: 31; CG: 31	42.39 ± 12.98	RA: 0.68 ± 0.17; CG: 0.48 ± 0.07	16.13% (N = 5)	12.9% (N = 4)	RA patients across all age groups revealed higher CIMT. As disease severity increased, CIMT increased considerably.
Targońska-Stȩpniak et al. (2019) [22]	Cross-sectional case-control	Poland	RA: 70; CG: 33	53.9 ± 13.1	RA: 0.83 ± 0.21; CG: 0.63 ± 0.09	N/A	N/A	Male RA patients had a significantly greater burden of atherosclerosis. Traditional CVD risk factors had a significant impact.
Galarza-Delgado et al. (2023) [23]	Cross-sectional case-control	Mexico	RA: 60; CG: 60	54.37 ± 8.88	RA: 0.75 (0.60-1.03); CG: 0.60 (0.50-0.70)	30% (N = 18)	11.7% (N = 7)	Subclinical atherosclerosis, including bilateral and carotid plaque, was more prevalent in RA patients during the first five years after diagnosis.
Arora et al. (2022) [24]	Cross-sectional case-control	India	RA: 64; CG: 64	46.7	RA: 0.74 ± 0.08; CG: 0.72 ± 0.07	N/A	N/A	Mean CIMT was higher in RA patients across all age groups, suggesting a higher subclinical atherosclerotic load.
Khaliq et al. (2023) [25]	Cross-sectional case-control	Pakistan	RA: 95; CG: 95	43.5 ± 12.8	RA: 0.55 ± 0.21; CG: 0.45 ± 0.19	N/A	N/A	When confounding variables (smoking, elevated LDL, total cholesterol, and triglycerides) were eliminated, CIMT in the RA group was 2.225 times greater than in the control group.
Gerasimova et al. (2023) [26]	Cross-sectional case-control	Russia	RA: 275; CG: 100	51 (47-54)	RA: 0.73 (0.60-0.81); CG: 0.73 (0.69-0.79)	27% (N = 47)	17% (N = 17)	This study suggests that RA patients, even at low CVD risk, are predisposed to subclinical atherosclerosis.
Wah-Suarez et al. (2019) [27]	Cross-sectional observational	Mexico	RA: 103; CG: 106	56.56 ± 9.6	RA (>0.9 mm), RC: 37.9% (N = 39); CG (>0.9 mm), RC: 15.1% (N = 15)	15.5% (N = 16)	6.6% (N = 7)	RA patients have a higher prevalence of subclinical atherosclerosis, evidenced by increased CIMT and higher rates of carotid plaques. RA-specific factors, such as chronic inflammation and disease activity, contribute to accelerated atherosclerosis.

Results of Individual Studies

Koren Krajnc et al. (2021) [20] reported an increase in CIMT measurements, with values of 0.688 ± 0.108 mm in the patient group and 0.604 ± 0.096 mm in the control group (p < 0.001) after 15 years from baseline. Similarly, Rajabzadeh et al. (2023) [21] found a significant difference in CIMT values, reporting 0.48 ± 0.07 mm for the control group and 0.68 ± 0.17 mm for the patient group (p < 0.001). In addition, Targońska-Stępniak et al. (2019) [22] showed that the mean CIMT value was significantly higher in male RA patients compared to male controls, as well as in female patients compared to female controls. Galarza-Delgado et al. (2023) [23] also found higher CIMT values in the patient group, with a median (IQR) of 0.75 (0.60-1.03) compared to controls, which had a median of 0.60 (0.50-0.70), with a statistically significant difference (p < 0.001).

Furthermore, according to Arora et al. (2022) [24], CIMT values in patients with RA were compared across different age groups with those of similar age groups in the control population. Significant statistical differences were observed in the 40-49 age group, where the values were 0.66 ± 0.07 mm for RA patients compared to 0.64 ± 0.06 mm in controls (p = 0.026). In the 50-59 age group, the CIMT was 0.80 ± 0.05 mm for RA patients versus 0.76 ± 0.05 mm for the control group (p = 0.047). Khaliq et al. (2023) [25] also found that the mean CIMT for the RA group was 0.55 ± 0.21 mm, while the control group had a mean CIMT of 0.45 ± 0.19 mm (p < 0.002), indicating a statistically significant difference. In contrast, Gerasimova et al. (2023) [26] reported a mean CIMT of 0.63 ± 0.81 mm in the RA group compared to 0.69 ± 0.79 mm in the control group, which was not statistically significant. Finally, Wah-Suarez et al. (2019) [27] found that a CIMT ≥ 0.09 cm was present in 50 out of 97 patients.

Discussion

RA affects mainly, but is not limited to, the joints, and systemic complications can affect the cardiovascular system with a subsequent increased risk of developing atherosclerosis. This systematic review studies the use of carotid ultrasound in the detection of subclinical atherosclerosis in patients with RA. The analysis of data from eight studies that were included provides insights into the clinical benefits of CIMT for the detection of carotid atheroma plaque. Our research indicates that an increase in CIMT measurement in patients with RA correlates with a higher risk of subclinical atherosclerosis. This study aligns with results presented from previous studies: a 2015 systematic review and meta-analysis reported RA patients had a statistically significantly greater CIMT [16,17,24,25], whereas aging is associated with a larger risk of atheroma plaque. Nevertheless, our study faces some limitations. Among them, heterogeneity between population groups (as some of the studies were focused only on women) and the degree of severity and time since diagnosis of RA are factors that could bias the results of our study. Our findings reinforce the need for larger, multicentric, randomized control trials, as most of the studies included were single-center with a small sample size population. Additionally, there is little to no use of carotid ultrasound in daily clinical practice; training is needed for healthcare providers on the importance and prognostic value of carotid ultrasound in the context of patients with RA.

## Conclusions

Our study emphasizes the importance and accessibility of using carotid ultrasound to evaluate patients with RA by measuring CIMT. As we know, patients with this condition are at a higher risk of developing subclinical atherosclerosis. Utilizing carotid ultrasound, a noninvasive and painless tool, offers a valuable opportunity for early detection, potentially delaying future clinical complications.
